# Dentoskeletal and tooth-size differences between Syrian and Hungarian adolescents with Class II division 1 malocclusion: a retrospective study

**DOI:** 10.1186/s13104-020-05115-0

**Published:** 2020-06-03

**Authors:** Alaa Al Ayoubi, Alireza Khandan Dezfully, Melinda Madléna

**Affiliations:** Department of Orthodontics and Pediatric Dentistry, Faculty of Dentistry, University of Szeged, Tisza Lajos körút 64-66, Szeged, 6720 Hungary

**Keywords:** Ethnic differences, Dentoskeletal structure, Tooth size, Class II division 1 malocclusion

## Abstract

**Objectives:**

Malocclusion features differ across various populations and ethnicities. At this time, no data are available regarding the dentofacial differences between Syrian and European adolescents with Class II division 1 malocclusion, which is one of the most frequently treated pathologies in orthodontic practice. The present combined cephalometric and tooth-size study aimed to compare the dentoskeletal and tooth-size characteristics of Syrian and Hungarian adolescents with Class II division 1 malocclusion.

**Results:**

Class II division 1 malocclusion in Hungarian adolescents was a sagittal discrepancy, while in Syrian adolescents, it was a result of excessive vertical development. Syrian adolescents had a significantly excessive vertical development when compared with Hungarian adolescents, regardless of sex *(p *< *0.01)*. Hungarian boys had significantly more protruded maxillae *(p *< *0.001)* and less retruded mandibles (*p *< *0.01)* when compared with Syrian boys, while Hungarian girls had significantly shorter mandibles relative to those of Syrian girls (*p *< *0.01)*. Syrian girls had significantly more protrusive lower incisors (*p *< *0.001)*, accompanied by significantly larger anterior tooth-size ratios when compared to Hungarian girls (*p *< *0.001)*. In conclusion, these findings underscore the importance of considering ethnic differences during orthodontic diagnosis and may have implications for optimizing orthodontic treatments in Syrian and Hungarian adolescents with Class II division 1 malocclusion.

## Introduction

Although several studies have reported ethnic differences in dentoskeletal structure and tooth-size characteristics [1–8], limited studies have investigated these differences in Class II division 1 (Class II/1) malocclusion [9–11]. With prevalences of 16% and 23.5% in Syrian and Hungarian adolescents, respectively [12, 13], this is one of the most frequently seen malocclusions in orthodontic practice.

Various factors cause Class II/1 malocclusion. However, conflicting results, possibly due to ethnic variations, have prevented their general characterization [9–11]. Likewise, tooth-size ratios show ethnic differences [4, 5]. In view of recent migration trends, especially from the Middle-East toward Europe, these findings underscore the need for updated comparisons of dentoskeletal and tooth-size characteristics across ethnicities [14]. Although some research has considered some dentoskeletal and tooth-size characteristics of Syrian populations [15, 16] or Hungarian populations [17, 18], no study has compared these characteristics between Syrian and European (Hungarian) adolescents.

Despite the evaluation of Class II/1 malocclusion in multiple studies, the following factors limit their results:

- The influence of skeletal age variations on results’ reliability has been neglected, since most Class II/1 malocclusion studies grouped patients by their chronological age [9–11, 19–23].
- While most studies selected patients with Class II/1 malocclusion based on their dental relationships (molar and/or incisor relationships) [9, 11, 20, 21, 23–25], these patients might have had either Class I or Class II skeletal pattern; studies on both skeletal and dental Class II/1 malocclusion are lacking [10, 22, 26].
- The dentoskeletal structure of individuals with Class II/1 malocclusion was analyzed during childhood [10, 11], adulthood [26], or by including subjects across a wide age-range—early childhood to adulthood [19–21]. However, most orthodontic patients seek treatment during adolescence, during which all treatment options and techniques are available.
- Limited studies have included cephalometric and tooth-size measurements in the same investigation [1]. This combination could comprehensively diagnose the malocclusion and elucidate the complex relationship between its components.

### Objective

This combined cephalometric and tooth-size study aimed to compare the skeletal morphology, dental position, and tooth size of skeletal age- and sex-matched Syrian and Hungarian adolescents with skeletal and dental Class II/1 malocclusion.

## Main text

### Materials and methods

#### Subjects

The sample size assumed a bilateral two-sample *t* test for assessment. According to previous similar dentoskeletal and tooth-size studies [5, 27], effect sizes were estimated from the SNGoMe angle 5.80° (± 5.78°) [27], and the upper central-incisor width 0.54 (± 0.52) mm [5], respectively. With a significance level alpha = 0.05 (two-sided) and 80% power, the required sample size was calculated to detect standardized effect sizes of 1.00 (5.80/5.78°) and 1.03 (0.54/0.52 mm) for dentoskeletal and tooth-size comparisons, respectively. Sample size calculation showed that 17 patients were required for each sex subgroup in each ethnic group [28]; lateral cephalograms and dental casts of 86 untreated patients with skeletal and dental Class II/1 malocclusion were thus included in this study. The study sample comprised two groups based on ethnicity—group 1, 43 Syrian patients selected from a private orthodontic office in Damascus, Syria; and group 2, 43 Hungarian patients selected from the Department of Orthodontics and Pediatric Dentistry, Faculty of Dentistry, University of Szeged, Hungary. Both groups included 24 girls and 19 boys. Each patient in group 1 was matched with a patient in group 2 by sex and skeletal age. To eradicate bias caused by growth variation, skeletal age was assessed from lateral cephalograms according to the method developed by Baccetti et al. [29]. Age and sex distributions of the study sample are summarized in Table 1.

**Table 1:** Age and sex distributions of the study groups

	Boys			Girls			Total
	Mean age ± S.D (y)	Age range (y)	Sample size (n)	Mean age ± S.D (y)	Age range (y)	Sample size (n)	
Syrians	14.1 ± 1.7	11.7–17.3	19	14.6 ± 1.4	11.9–17.1	24	43
Hungarians	14.0 ± 2.0	11.4–17.6	19	14.6 ± 1.8	11.3–17.7	24	43
Total			38			48	86

The inclusion criteria were as follows:

- Age, 12–17 years.
- Syrian ethnicity for group 1; Hungarian ethnicity for group 2.
- Overjet > 4 mm without upper incisor retroclination (U1/NA angle ≥ 22°).
- Half-unit or greater bilateral distal occlusion with permanent dentition.
- ANB angle > 4° with a convex facial profile.
- No extractions, interproximal restorations, or conditions affecting the mesio-distal teeth diameter.

The exclusion criteria were patients with history of orthodontic treatment, trauma, or craniofacial syndromes. The Human Investigation Review Board at the University of Szeged (151/2018-SZTE) approved this retrospective cross-sectional study. The participants or their parent/legal guardian provided written informed consent to participate in this study.

#### Cephalometric measurements

Pretreatment lateral cephalograms were obtained for each patient in both ethnic groups using the same protocol (head in natural position and teeth in maximal intercuspation). Magnification was corrected to 1:1 since the cephalograms were acquired with two different machines. The cephalometric measurements used herein were derived from the analyses developed by Jarabak, Steiner, and the University of Bonn [30–32]. Definitions of the cephalometric measurements are presented in Additional file 1: Table S1. Landmarks and reference lines are shown in Additional file 2: Figure S1. A special orthodontic software (OnyxCeph3™, Image Instruments GmbH, Chemnitz, Germany) was used by one investigator to digitize and analyze all lateral cephalograms.

#### Dental-cast measurements

The teeth in both arches—from the right first permanent molar to the left first permanent molar—were measured at the largest mesio-distal dimension [33], to the nearest 0.01-mm, by one investigator using a universal digital caliper (MIB Messzeuge GmbH, Spangenberg, Germany). Bolton’s overall ratio (∑widths of the mandibular 6–6 / ∑widths of the maxillary 6–6 × 100) [34] and Bolton’s anterior ratio (∑widths of the mandibular 3–3 / ∑widths of the maxillary 3–3 × 100) [34] were calculated and used in statistical analyses.

#### Method error

To establish intra-examiner reliability, measurements of 20 randomly selected cephalograms and casts were replicated several weeks later by the same investigator. Dahlberg’s formula [35] showed random errors ≤ 0.40 mm and ≤ 0.44° for linear and angular cephalometric variables, respectively, and ≤ 0.17 mm for tooth-size measurements. Paired sample *t*-tests showed no systematic error (p > 0.05). Intraclass correlation coefficients were > 0.95.

To establish inter-examiner reliability, measurements of 20 randomly selected cephalograms and casts were replicated again by another investigator. Random errors were ≤ 0.46 mm and ≤ 0.48° for linear and angular cephalometric variables, respectively, and ≤ 0.33 mm for tooth-size measurements. Systematic error was absent (p > 0.05). Intraclass correlation coefficients were > 0.93.

#### Statistical analyses

Descriptive statistics of each variable were calculated using SPSS software 24.0 (SPSS Inc., Chicago, USA). Intergroup comparisons were performed using *t*- or Mann–Whitney *U*-tests, depending on data normality (according to Shapiro–Wilk’s test). For normally-distributed data, two-sample or Welch’s *t*-tests were used depending on equality of variance (according to *F*-test). The level of statistical significance was set at 0.05.

### Results

Results for sex-based comparisons between the two ethnic groups are presented in Table 2.

**Table 2:** Sex‐based comparison of cephalometric measurements and tooth-size ratios between the two ethnic groups

	Boys					Girls				
	Syrians (*n *= 19) Mean ± S.D	Hungarians (*n *= 19) Mean ± S.D	95% CI of Mean difference		*p*-value	Syrians (*n *= 24) mean ± S.D	Hungarians (*n *= 24) Mean ± S.D	95% CI of Mean difference		*p*-value
			L	U				L	U	
Cephalometric measurements ∂										
Skeletal measurements										
Sagittal values										
SNA (°)	79.70 ± 2.72	83.43 ± 2.79	− 5.54	− 1.91	*< 0.001*	81.06 ± 2.50	80.69 ± 3.49	− 1.40	2.13	0.679
SNB (°)	73.81 ± 2.93	76.73 ± 2.82	− 4.81	− 1.03	*0.003*	74.05 ± 2.85	74.24 ± 3.62	− 2.09	1.70	0.840
ANB (°)	5.89 ± 1.46	6.70 ± 1.19	− 1.69	0.06	0.068	7.01 ± 1.75	6.45 ± 1.56	− 0.41	1.52	0.249
ANS-PNS (mm)	56.44 ± 4.95	56.68 ± 3.12	− 2.97	2.49	0.859	55.00 ± 4.04	53.39 ± 3.15	− 0.50	3.71	0.132
Go-Gn (mm)	73.13 ± 6.18	71.38 ± 5.05	− 1.96	5.47	0.345	71.93 ± 4.58	67.77 ± 4.50	1.52	6.79	*0.003*
Vertical values										
ArGoMe (°)	124.08 ± 7.88	119.11 ± 6.25	0.30	9.66	*0.038*	125.29 ± 9.62	120.18 ± 6.67	0.30	9.92	*0.038*
∑ Bjork (°)	398.33 ± 5.75	392.16 ± 5.85	2.35	9.99	*0.002*	400.14 ± 6.43	394.53 ± 6.75	1.78	9.44	*0.005*
Ar-Go (mm)	41.56 ± 5.11	44.48 ± 4.70	− 6.15	0.30	0.074	42.01 ± 4.81	39.98 ± 4.17	− 0.58	4.65	0.124
SN/GoMe (°)	38.33 ± 5.76	32.16 ± 5.85	2.35	9.99	*0.002*	40.14 ± 6.43	34.53 ± 6.75	1.78	9.44	*0.005*
S-Go:N-Me (%)	61.64 ± 4.09	66.71 ± 4.51	− 7.90	− 2.24	*0.001*	60.62 ± 4.69	64.47 ± 5.42	− 6.80	− 0.90	*0.012*
Dental measurements										
U1/NA (°)	26.97 ± 4.18	26.98 ± 3.71	− 2.62	2.58	0.989	26.44 ± 2.60	28.16 ± 4.32	− 3.81	0.36	0.102
L1/NB (°)	29.37 ± 5.56	28.80 ± 5.23	− 2.99	4.11	0.750	35.45 ± 4.34	27.50 ± 6.05	4.89	11.01	*< 0.001*
U1-NA (mm)	5.86 ± 2.06	4.90 ± 2.11	− 0.42	2.33	0.166	6.21 ± 2.15	5.39 ± 2.29	− 0.47	2.11	0.208
L1-NB (mm)	8.22 ± 2.03	6.88 ± 2.08	− 0.01	2.69	0.052	9.73 ± 1.96	5.52 ± 1.88	3.09	5.32	*< 0.001*
Tooth-size ratios ∂										
Anterior ratio (%)	80.55 ± 2.95	79.42 ± 2.08	− 0.57	2.81	0.186	80.81 ± 2.60	77.89 ± 2.42	1.46	4.38	*< 0.001*
Overall ratio (%)	92.74 ± 1.80	92.79 ± 2.33	− 1.42	1.32	0.941	92.92 ± 1.65	91.87 ± 1.98	0.00	2.11	0.051

Italic values indicate significance of *p* value (*p* < 0.05)

∂ *t*-tests for independent variables

*S.D* Standard deviation, *CI* Confidence interval

Results for overall comparisons between the two ethnic groups are presented in Additional file 3: Table S2.

#### Cephalometric comparisons

**Fig. 1:**
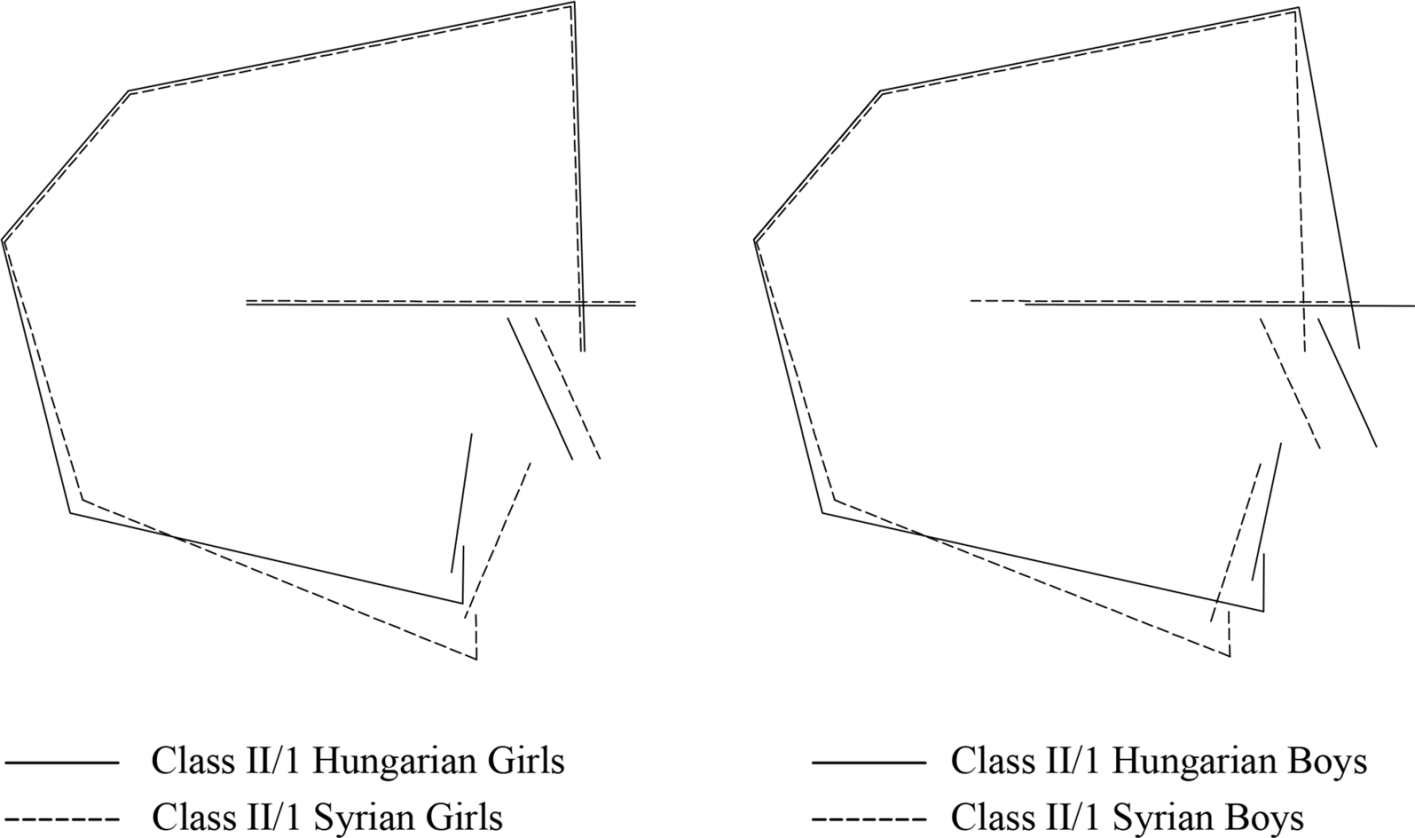
Sex-based comparisons between Syrian and Hungarian adolescents with Class II/1 malocclusion

Sagittal comparisons revealed that Hungarian boys had significantly more protruded maxillae (SNA) than their Syrian counterparts (*p *< 0.001), while Syrian boys had significantly more retruded mandibles (SNB) (*p *< 0.01). Hungarian girls had significantly smaller mandibular lengths (Go-Gn) than their Syrian counterparts (*p *< 0.01) (Fig. 1).

Vertical measurements (ArGoMe, ∑Bjork, SN/GoMe, and S-Go:N-Me) showed significant differences (*p *< 0.01) between Syrian and Hungarian adolescents, regardless of sex, indicating a hyperdivergent facial pattern in Syrian ethnicity (Additional file 3: Table S2).

Dental measurement results revealed that Syrian girls had significantly more protruded lower incisors (L1/NB and L1-NB) (*p *< 0.001) than Hungarian girls. These observations did not extend to the male populations (Table 2).

#### Tooth-size comparisons

Syrian girls had significantly greater anterior tooth-size ratios than Hungarian girls (*p *< 0.001). Syrian and Hungarian boys showed no such difference (Table 2).

### Discussion

This study evaluated the effects of ethnic variations on dentoskeletal and tooth-size characteristics of Syrians and Hungarians adolescents with Class II/1 malocclusion. As most dentoskeletal variations can be ascribed to sex, age (maturity stage), and ethnicity [9], and most tooth-size variations can be ascribed to sex and ethnicity [4, 5], this study compared sex- and skeletal age-matched individuals to yield clear conclusions on the impact of ethnicity on dentoskeletal and tooth-size characteristics.

Skeletal comparisons showed that sagittal measurements significantly differed between the two groups—Hungarian boys had more protruded maxillae compared to Syrian boys, while Hungarian girls had significantly shorter mandibles than Syrian girls (Fig. 1). These observations in Hungarian adolescents support those of a previous study [19], which found Class II/1 Caucasian boys with maxillary protrusion and Class II/1 Caucasian girls with shorter-than-average mandibular length. Although Syrian adolescents had relatively normal maxillary position and mandibular length as compared with Hungarian adolescents, their mandibles were posteriorly positioned. This was evident in Syrian boys with significantly smaller SNB angles than Hungarian boys who had relatively normal mandibular position. In contrast, Syrian girls also had restricted mandibles; however, the diminished mandibular sizes of Hungarian girls were pronounced enough to cause a non-significant difference in SNB measurements between the two female populations. Non-Caucasian studies reported similar findings of normal maxillary position and retruded mandible in Class II/1 Syrian subjects [20, 22, 23, 26]. Contrarily, some Caucasian studies reported varying findings with normally positioned mandibles and protruded maxillae [24, 25]. The involvement of different ethnicities across various studies may explain the inconsistent findings.

Many authors have recognized the importance of excessive vertical development in the determination of mandibular position [10, 20, 26]. Thus, retruded mandibles of Syrian adolescents in this study can be explained with respect to the vertical plane: Syrian adolescents, in comparison to Hungarian adolescents, regardless of sex, had hyperdivergent facial patterns (Fig. 1). This finding agrees with those of other studies that compared non-Caucasian and Caucasian Class II patients [9, 10, 27].

The position of dentition, relative to the skeletal structure, is another important aspect of Class II/1 malocclusion. The only significant difference in the dental position found in this study was related to the lower incisors and was evident among girls. Syrian girls had more protrusive lower incisors than Hungarian girls. Status on the position of lower incisors in patients with Class II/1 malocclusion remains unclear. Most non-Caucasian studies have reported protruded lower incisors [20–22, 26], and two comparative studies [9, 10] support ethnic variations in lower incisor position between non-Caucasian and Caucasian populations. Since a tooth-size excess with space limitation might cause incisor protrusion, the larger anterior tooth-size ratios of Syrian girls relative to Hungarian girls might further account for their lower incisor protrusion. This is because the large anterior tooth-size ratios of Syrian girls reflect a relative tooth-size excess in the lower anterior region. The significant difference in anterior tooth-size ratio between Syrian and Hungarian girls can be attributed to ethnic variations [4, 5].

#### Clinical implications

First, because Class II/1 malocclusion in Hungarian adolescents represents sagittal discrepancy (protruded maxillae in boys; short mandibles in girls), treatment strategies should aim to inhibit maxillary growth using extra-oral forces in Hungarian boys and enhance mandibular growth using functional appliances in Hungarian girls. Second, since Syrian adolescents had considerable vertical tendencies and most orthodontic treatment mechanics tend to open the bite, greater care should be exercised to control the vertical dimension when treating Syrian adolescents compared with Hungarian adolescents. Treatment strategies for Class II/1 Syrian adolescents should aim to alter the extent and direction of vertical facial growth and prevent posterior mandibular rotation. Finally, Class II/1 Syrian girls exhibited more protrusive lower incisors with relative tooth-size excess in the lower anterior segment, which affects treatment objectives regarding the optimal final position of these teeth. Therefore, a more interproximal reduction might be a better indication for Syrian girls than Hungarian girls, if the treatment decision is to decrease protrusion. Contrarily, protruded lower incisors, more than the standard levels, might be acceptable outcomes in Syrian girls.

## Conclusions

Class II/1 treatment strategies for Hungarian adolescents are not applicable to Syrian adolescents, because.

- Class II/1 malocclusion reflects sagittal discrepancy in Hungarian adolescents (protruded maxillae in boys; short mandibles in girls), while it was caused by excessive vertical growth among Syrian adolescents, regardless of sex.
- Class II/1 Syrian girls have more protrusive lower incisors with a relative tooth-size excess in the lower anterior region compared with Class II/1 Hungarian girls.

Thus, even among patients with the same malocclusion type, dentoskeletal and tooth-size characteristics can vary with ethnicity. Hence, orthodontists should be aware of this variation to optimize their differential diagnosis and treatment planning.

## Limitations

Although sample size estimation showed sufficient sample sizes, they were relatively small. This was the major drawback of the present study; therefore, the results should be interpreted with caution, and additional studies with larger sample sizes are warranted.

## Supplementary information

**Additional file 1: Table S1.** Definitions of the cephalometric measurements used in this study.

**Additional file 2: Figure S1.** Landmarks and reference lines used in this study.

**Additional file 3: Table S2.** Overall comparison of cephalometric measurements and tooth-size ratios between the two ethnic groups.

## Data Availability

The datasets generated and analyzed during the current study are available from the corresponding authors on reasonable request.

## Abbreviation

Class II/1: Class II division 1
