# Supplementary material for: Dentoskeletal and tooth-size differences between Syrian and Hungarian adolescents with Class II division 1 malocclusion: a retrospective study
Source: BMC Res Notes. 2020 Jun 3;13:270. doi: 10.1186/s13104-020-05115-0 (PMC7268623; doi:10.1186/s13104-020-05115-0)
Supplement: Supplementary file 1 — Additional file 1: Table S1. Definitions of the cephalometric measurements used in this study. [file 13104_2020_5115_MOESM1_ESM.docx]

**Additional file 1: Table S1.** Definitions of the cephalometric measurements used in this study.

| Skeletal measurements |  |
| --- | --- |
| Sagittal values |  |
| 1. SNA | Angle formed by connecting the S-N plane to A point. |
| 1. SNB | Angle formed by connecting the S-N plane to B point. |
| 1. ANB | Angle formed by subtracting SNB angle from SNA angle. |
| 1. ANS-PNS | Distance between ANS point (anterior nasal spine) and PNS point (posterior nasal spine) determining the length of the maxilla. |
| 1. Go-Gn | Distance from Gn point (Gnathion) to Go point (Gonion) determining the length of the mandible. |
| Vertical values |  |
| 1. ArGoMe (Gonial angle) | Angle between the horizontal and the vertical part of the lower jaw, formed by the reference lines Ar-Go and Go-Me. |
| 1. ∑ Bjork | Sum of the angles NSAr, SArGo and ArGoMe, formed by the reference lines N-S, S-Ar, Ar-Go and Go-Me. |
| 1. Ar-Go | Distance from Ar point (Artikulare) to Go point determining the length of the mandibular ramus. |
| 1. SNGoMe | Angle formed by the reference lines S-N and Me-Go, determines the inclination of the mandibular plane to the anterior cranial base. |
| 1. S-Go:N-Me | Ratio between anterior facial height (distance from N point to Me point) and posterior facial height (distance from S point to Go point). |
| Dental measurements |  |
| 1. U1/NA | Angle formed by the long axis of the most prominent upper incisor and the line N-A. |
| 1. L1/NB | Angle formed by the long axis of the most prominent lower incisor and the line N-B. |
| 1. U1-NA | Distance from the labial outline of the crown of the most prominent upper incisor to the line N-A. |
| 1. L1-NB | Distance from the labial outline of the crown of the most prominent lower incisor to the line N-B. |
