# Supplementary figures and images for: Dentoskeletal and tooth-size differences between Syrian and Hungarian adolescents with Class II division 1 malocclusion: a retrospective study
Source: BMC Res Notes. 2020 Jun 3;13:270. doi: 10.1186/s13104-020-05115-0 (PMC7268623; doi:10.1186/s13104-020-05115-0)

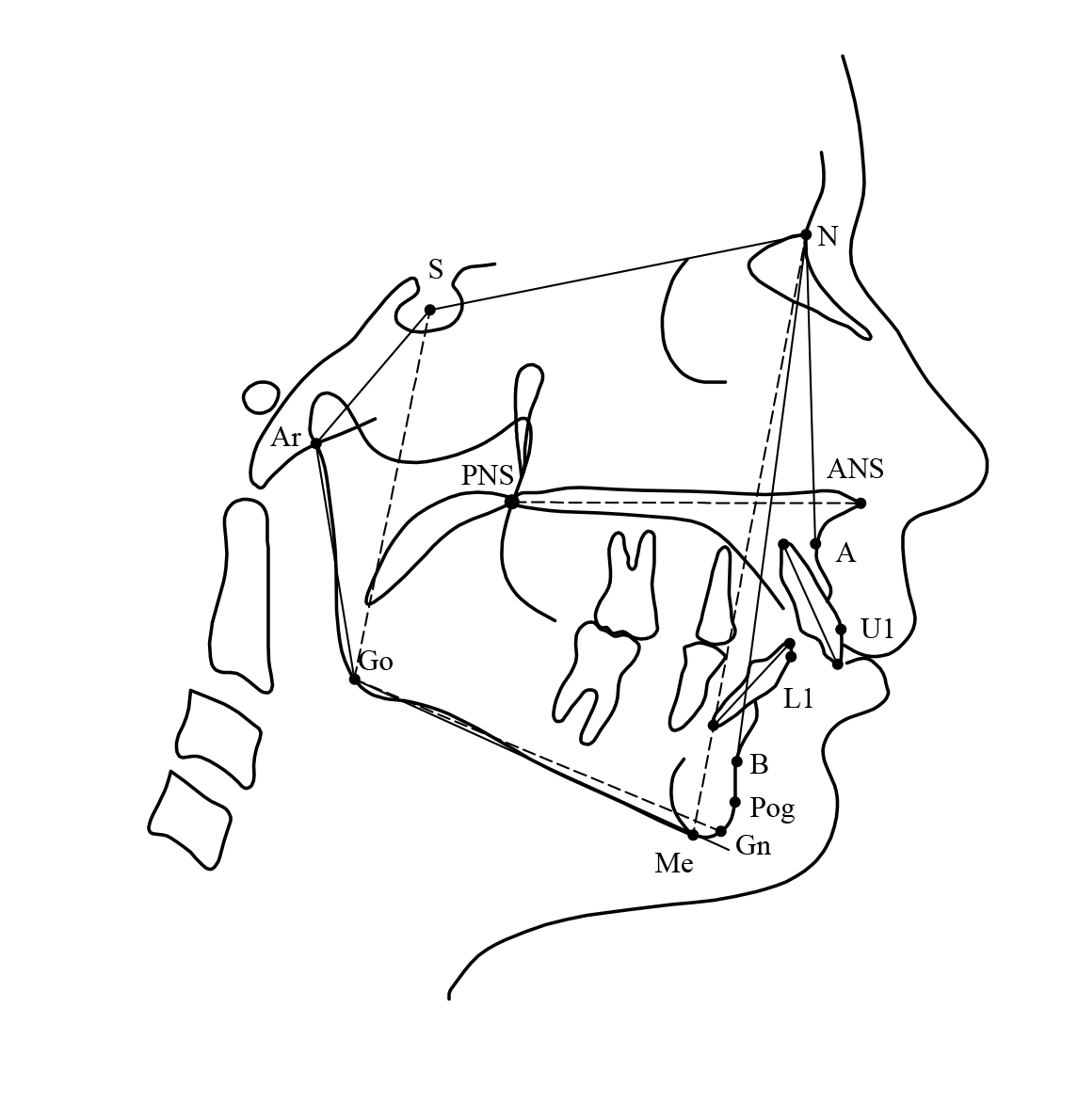

Supplement: Supplementary file 2 — Additional file 2: Figure S1. Landmarks and reference lines used in this study. [file 13104_2020_5115_MOESM2_ESM.tiff]
