# Supplementary material for: Dentoskeletal and tooth-size differences between Syrian and Hungarian adolescents with Class II division 1 malocclusion: a retrospective study
Source: BMC Res Notes. 2020 Jun 3;13:270. doi: 10.1186/s13104-020-05115-0 (PMC7268623; doi:10.1186/s13104-020-05115-0)
Supplement: Supplementary file 3 — Additional file 3: Table S2. Overall comparison of cephalometric measurements and tooth-size ratios between the two ethnic groups. [file 13104_2020_5115_MOESM3_ESM.docx]

**Additional file 3: Table S2.** Overall comparison of cephalometric measurements and tooth-size ratios between the two ethnic groups.

|  | **Syrian adolescents**  **(n = 43)** | **Hungarian adolescents**  **(n = 43)** | **95% CI of Mean/Median Difference** | | **P-value** |
| --- | --- | --- | --- | --- | --- |
|  |  |  | **L** | **U** |  |
| **Cephalometric measurements** |  |  |  |  |  |
| Skeletal measurements |  |  |  |  |  |
| Sagittal values |  |  |  |  |  |
| SNA (°) (Mean ± S.D) **∂** | 80.46 ± 2.66 | 81.90 ± 3.45 | -2.76 | -0.12 | **0.033** |
| SNB (°) (Mean ± S.D) **∂** | 73.94 ± 2.86 | 75.34 ± 3.49 | -2.76 | -0.03 | **0.045** |
| ANB (°) (Median / IQR) **†** | 6.48 / 2.45 | 6.18 / 1.78 | -0.56 | 0.74 | 0.704 |
| ANS-PNS (mm) (Median / IQR) **†** | 54.43 / 5.17 | 54.78 / 5.55 | -2.08 | 1.33 | 0.749 |
| Go-Gn (mm) (Mean ± S.D) **∂** | 72.46 ± 5.31 | 69.37 ± 5.03 | 0.87 | 5.31 | **0.007** |
| Vertical values |  |  |  |  |  |
| ArGoMe (°) (Mean ± S.D) **∂** | 124.76 ± 8.81 | 119.71 ± 6.44 | 1.74 | 8.37 | **0.003** |
| ∑ Bjork (°) (Mean ± S.D) **∂** | 399.34 ± 6.13 | 393.48 ± 6.40 | 3.17 | 8.55 | **<0.001** |
| Ar-Go (mm) (Median / IQR) **†** | 41.48 / 7.77 | 41.34 / 5.55 | -1.62 | 2.41 | 0.650 |
| SN/GoMe (°) (Mean ± S.D) **∂** | 39.34 ± 6.14 | 33.48 ± 6.40 | 3.17 | 8.55 | **<0.001** |
| S-Go:N-Me (%) (Mean ± S.D) **∂** | 61.07 ± 4.41 | 65.46 ± 5.11 | -6.44 | -2.34 | **<0.001** |
| Dental measurements |  |  |  |  |  |
| U1/NA (°) (Median / IQR) **†** | 25.96 / 4.61 | 26.60 / 7.24 | -0.75 | 2.33 | 0.409 |
| L1/NB (°) (Mean ± S.D) **∂** | 32.76 ± 5.74 | 28.08 ± 5.67 | 2.24 | 7.13 | **<0.001** |
| U1-NA (mm) (Mean ± S.D) **∂** | 6.05 ± 2.09 | 5.17 ± 2.20 | -0.04 | 1.80 | 0.061 |
| L1-NB (mm) (Mean ± S.D) **∂** | 9.06 ± 2.11 | 6.12 ± 2.06 | 2.05 | 3.84 | **<0.001** |
|  |  |  |  |  |  |
| **Tooth-size ratios** |  |  |  |  |  |
| Anterior ratio (%) (Mean ± S.D) ∂ | 80.69 ± 2.73 | 78.57 ± 2.38 | 1.03 | 3.22 | **<0.001** |
| Overall ratio (%) (Mean ± S.D) ∂ | 92.84 ± 1.70 | 92.28 ± 2.16 | -0.27 | 1.40 | 0.180 |

**∂** *t*-tests for independent variables

**†** Mann-Whitney *U*-test for independent variables

CI=Confidence interval, S.D=Standard deviation, and IQR=Interquartile range
